# Associations of Human Milk Oligosaccharides with Infant Brain Tissue Organization and Regional Blood Flow at 1 Month of Age

**DOI:** 10.3390/nu14183820

**Published:** 2022-09-16

**Authors:** Paige K. Berger, Ravi Bansal, Siddhant Sawardekar, Chloe Yonemitsu, Annalee Furst, Hailey E. Hampson, Kelsey A. Schmidt, Tanya L. Alderete, Lars Bode, Michael I. Goran, Bradley S. Peterson

**Affiliations:** 1 Department of Pediatric Newborn Medicine, Brigham and Women’s Hospital, Harvard Medical School, Boston, MA 02115, USA; 2 Department of Pediatrics, The Saban Research Institute, Children’s Hospital Los Angeles, Los Angeles, CA 90027, USA; 3 Department of Pediatrics and Mother-Milk-Infant Center of Research Excellence, University of California, San Diego, CA 92093, USA; 4 Department of Integrative Physiology, University of Colorado Boulder, Boulder, CO 80309, USA

**Keywords:** breastfeeding, magnetic resonance imaging, brain development, infancy, oligosaccharides, lactation

## Abstract

Animal studies have shown that human milk oligosaccharides (HMOs) are important in early brain development, yet their roles have not been assessed in humans. The purpose of this study was to determine the associations of HMOs with MRI indices of tissue microstructure and regional cerebral blood flow (rCBF) in infants. Mother–infant pairs (N = 20) were recruited at 1 month postpartum. Milk was assayed for the concentrations of the HMOs 2′-fucosyllactose (2′FL), 3-fucosyllactose (3FL), 3′-sialyllactose (3′SL), and 6′-sialyllactose (6′SL). Diffusion and arterial spin labeling measures were acquired using a 3.0-Tesla MRI scanner. Multiple linear regression was used to assess the voxel-wise associations of HMOs with fractional anisotropy (FA), mean diffusivity (MD), and rCBF values across the brain. After adjusting for pre-pregnancy BMI, sex, birthweight, and postmenstrual age at time of scan, a higher 2′FL concentration was associated with reduced FA, increased MD, and reduced rCBF in similar locations within the cortical mantle. Higher 3FL and 3′SL concentrations were associated with increased FA, reduced MD, and increased rCBF in similar regions within the developing white matter. The concentration of 6′SL was not associated with MRI indices. Our data reveal that fucosylated and sialylated HMOs differentially associate with indices of tissue microstructure and rCBF, suggesting specific roles for 2′FL, 3FL, and 3′SL in early brain maturation.

## 1. Introduction

Human milk is the gold standard for infant nutrition, as research suggests that a greater frequency and duration of breastfeeding benefits infant health [1,2]. However, evidence in support of the benefits of breastfeeding for some infant outcomes has been inconsistent [3], perhaps in part because of differences in milk composition over the course of lactation, between women, and across populations. While human milk composition is complex, and its determination presents methodological challenges for research, it is critically important to consider the variation in the concentrations of milk components to fully understand the benefits of breastfeeding as part of a biological system, the mother–milk–infant ‘triad’ [4].

Human milk oligosaccharides (HMOs) are structurally diverse, complex carbohydrates that may confer some of the proposed benefits of human milk on infant outcomes [5,6]. HMO biosynthesis follows a basic blueprint that begins with a lactose molecule that is elongated and either fucosylated or sialylated to create various subgroups [7]. These slight structural differences impart diverse physiological functions on HMOs in infant health [7]. Our research team reported that higher concentrations of the HMO 2′-fucosyllactose (2′FL) in the maternal milk at 1 month from delivery predicted better cognitive development of the infant at 24 months of age [6]. Animal studies have shown that feeding 2′FL during lactation enhanced learning and memory in rats, whereas feeding the sialylated HMOs 3′-sialyllactose (3′SL) and 6′-sialyllactose (6′SL) improved cognitive performance in piglets [8,9]. HMOs are thought to promote early brain development through at least two mechanisms: (1) HMOs are prebiotics that shape the development of the gut microbiome and its production of metabolites that affect brain functions [10,11]; and (2) HMOs are a source of sialic acid (SA), an essential nutrient in ganglioside formation and myelination, key components of the cortical gray matter and developing white matter [9]. The specific effects of individual HMOs on infant brain development, however, are unknown.

Therefore, we performed a study using magnetic resonance imaging (MRI) to determine how individual HMO concentrations in maternal milk were associated with measures of brain tissue organization and regional cerebral blood flow (rCBF) in infants at 1 month of age. The MRI modalities used to assess brain development included: (1) diffusion tensor imaging (DTI), which measures the diffusion of water as influenced by characteristics of brain tissue microstructure; and (2) arterial spin labeling (ASL), which quantifies rCBF, a surrogate measure of cellular metabolism [12,13,14].

## 2. Materials and Methods

### 2.1. Subjects

Twenty mother–infant pairs were recruited from maternity clinics in Los Angeles County as part of a larger cohort study, and the inclusion criteria have been previously described [15]. Briefly, the mothers were included if they were ≥18 years old at delivery, had given birth to a full-term singleton newborn, were breastfeeding, and were enrolled within 1 month from delivery [15]. Mothers were excluded if they reported medications or medical conditions that could affect their physical or mental health, nutrition, or metabolism, used tobacco or recreational drugs, or had a clinical diagnosis of fetal abnormalities [15]. The Institutional Review Board of Children’s Hospital Los Angeles approved all procedures (Protocol ID: CHLA-18-00576). The participants provided written informed consent prior to data collection.

### 2.2. Study Design

The mother–infant pairs were assessed at 1 month postpartum. Historical health-related information was collected. The mothers pumped a full expression of breast milk that was analyzed for individual HMO concentrations. The infants underwent MRI scanning at approximately 1 month postpartum to assess brain tissue microstructure and rCBF.

### 2.3. Human Milk Collection and Analysis

Breast milk was collected and analyzed following standard procedures [6]. The mothers were asked to avoid eating and drinking for 1 h and feeding or pumping breast milk for 1.5 h before collection. Mothers were encouraged to pump the entire content of a single breast expression to ensure the collection of fore, mid, and hind milk to standardize milk collection as much as possible [16,17]. Aliquots were stored at −80 °C until HMO characterization at the University of California San Diego. Raffinose was added to each sample as an internal standard for absolute quantification. HMOs were isolated with high-throughput solid-phase extraction, fluorescently labeled, and measured using HPLC [18]. Nineteen HMOs were quantified based on standard retention times and mass spectrometric analysis, which accounted for >90% of total HMO composition. Our initial focus, however, was on the most abundant fucosylated and sialylated HMOs reported to influence brain development in animals and infants: 2′FL, 3FL, 3′SL, and 6′SL [6,9,19,20,21]. The secretor status was defined by the presence or near absence of the HMOs 2′FL or lacto-N-fucopentaose I [22].

### 2.4. MRI Scanning Procedures

MRI data were collected on a 3 Tesla Philips Achieva MRI scanner equipped with a 32-channel receive-only head coil. All data were acquired without sedation or contrast agent. The mothers and the staff lulled the infants to sleep with feeding and swaddling. The scans were visually monitored and repeated for any visible motion. The detailed image quality was assessed during preprocessing within 48 h of scan acquisition.

### 2.5. Image Processing

DTI. The template brain was selected using a 2-step procedure to find the brain that was morphologically representative of all brains in our sample, as described in Supplementary Material File S1 [23,24,25,26,27,28,29,30,31]. We instituted quality assurance procedures to exclude datasets with excessive motion, also described in Supplemental Material File S1 [23,24,25,26,27,28,29,30,31]. We estimated the diffusion tensor (D) at each voxel of the pre-processed DTI data in DSI Studio (RRID:SCR_009557) and computed the scalar indices fractional anisotropy (FA), mean diffusivity (MD), axial diffusivity (AD), and radial diffusivity (RD). These maps were edited to remove nonbrain tissue and then were threshed to remove background noise. Edited MD maps were used to mask out nonbrain tissue in the FA, AD, and RD maps. The edited maps for each infant were coregistered using a rigid body transformation (3 translations and 3 rotations) to the infant’s T1-weighted (T1w) anatomical image, which were in turn used as an intermediary source to coregister the maps for each infant to the final template brain. The FA, AD, MD, and RD maps were then smoothed using a Gaussian kernel with FWHM = 4 mm.

ASL. We aligned the rCBF brain images and the M_0_WM_ image to the first rCBF image for each participant in native imaging space to correct for head motion. Images with >0.5 mm inter-frame motion were excluded from further processing. We spatially smoothed the coregistered rCBF images using a Gaussian kernel of FWHM = 4 mm to improve the signal-to-noise ratio while avoiding loss of spatial precision in locating our effects of interest. We generated a brain mask for each infant using the mean rCBF image. We used in-house software to construct for each infant a voxel-wise map of rCBF from the ASL time series and M_0_WM_ image: (1) We pair-wise subtracted the control images from the labeled images; (2) Perfusion-weighted images were calculated by pair-wise subtraction of the control and labeled images, followed by averaging across the imaging time series. The perfusion map (rCBF) was calculated using a single compartment model [27]. We used a linear transformation with 6 degrees of freedom to coregister the quantitative rCBF maps for each infant to their T1-weighted anatomical images, which were in turn used as an intermediary source to coregister the rCBF images for each infant to the final brain template (Supplementary Material File S1 [23,24,25,26,27,28,29,30,31]).

### 2.6. Statistical Analysis

Descriptive statistics are presented as mean ± standard deviation (SD) for continuous variables and as frequency (percentage) for categorical variables. Normal distribution and homogeneity of variances were confirmed by the Shapiro–Wilks W and Levene’s tests. Values >3 SD from the mean were identified as outliers. We used multiple linear regression applied to each voxel of the image to assess the significance of the correlation coefficient for the concentration (nmol/mL) of each HMO (2′FL, 3FL, 3′SL, 6′SL) and total HMO-bound Sia with FA, MD, and rCBF values. We assessed the significance first in the total cohort (N = 20) and then in infants of secretor mothers only (N = 17), as secretor mothers have an active secretor locus that encodes for a functional fucosyltransferase 2 enzyme and produce higher concentrations of alpha-1-2-fucosylated HMOs such as 2′FL relative to those classified as non-secretors. Pre-pregnancy BMI, postmenstrual age (PMA) at the time of the MRI scan, birthweight, and sex were included as covariates in all analyses [6,32]. We used the Benjamini–Yekutieli procedure for False Discovery Rate to correct the *p*-values for the number of statistical comparisons in the regressions modeling each HMO exposure variable [33]. The corrected *p*-values were color-coded and displayed on the template brain. All statistical maps were constructed using in-house software. The regions of interest were identified using an age-specific DTI atlas for the infant brain [34]. With a given sample size of 20, alpha-level of 0.05, and medium effect size for correlation coefficient, the study had a power of 0.69 to detect real effects (G*Power, version 3.1.9.6).

## 3. Results

**Figure 1:**
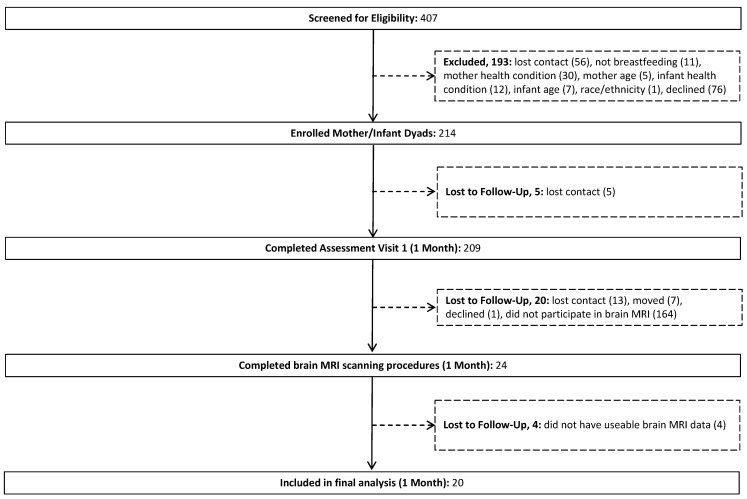
Participant flow chart.

The characteristics of the mother–infant dyads are shown in Table 1. Of the participants enrolled in the larger study, 20 mothers whose infants had usable MRI data at 1 month of age were included in the analysis (Figure 1). All mothers self-identified as Hispanic. By design, the infants were born full-term with normal birthweight (i.e., ≥2500 g). The proportions of males and females were similar. The mothers reported 7.1 ± 1.6 feedings per day, defined as exclusive milk feeding for infants at 1 month of age. Eighty-five percent of the mothers were HMO secretors.

### 3.1. HMO 2′FL Exposure at 1 Month of Age and Newborn MRI Measures

**Figure 2:**
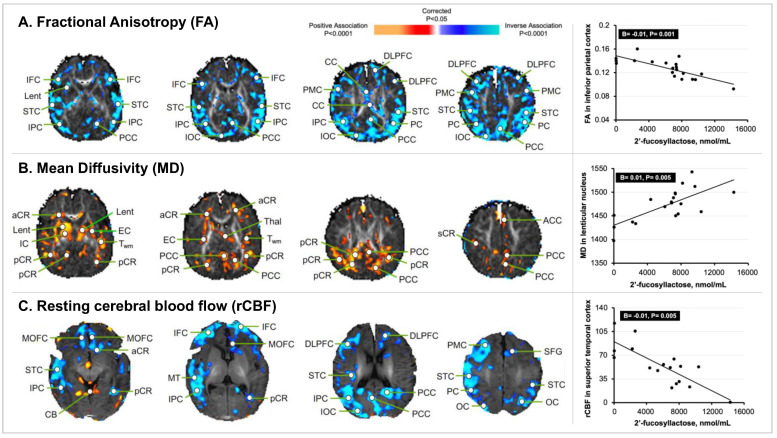
**Statistical maps of 2′FL exposure at 1 month of age with newborn MRI measures.** The statistical significance of the associations of 2′FL exposure with measures of brain tissue microstructure and rCBF at each point on the surface of the brain is color-coded, with warm colors representing significant positive associations, and cool colors representing significant inverse associations. Only the *p*-values that survived the FDR correction are plotted. (**A**) Concentrations of 2′FL inversely associated with the FA values throughout much of the cortical mantle. The scatterplot highlights the significant inverse association of 2′FL exposure with FA values located in the right IPC. (**B**) Concentrations of 2′FL positively associated with the MD values in the posterior gray matter, posterior white matter, and subcortical gray matter nuclei. The scatterplot highlights the significant positive association of 2′FL exposure with the MD values located in the Lent. (**C**) Concentrations of 2′FL inversely associated with the rCBF values in the cortical gray matter in similar locations as those of the FA values. The scatterplot highlights the significant inverse association of 2′FL exposure with rCBF values located in the left STC. Abbreviations: ACC, anterior cingulate cortex; aCR, anterior corona radiata; BG, basal ganglia; CB, cerebellum; CG, cingulum; CC, corpus callosum; DLPFC, dorsolateral prefrontal cortex; EC, external capsule; FC, frontal cortex; F_WM_, frontal white matter; IFC, inferior frontal cortex; IOC, inferior occipital cortex; IPC, inferior parietal cortex; IN, insula; IC, internal capsule; LOFC, lateral orbitofrontal cortex; lent, lenticular nucleus; MOFC, medial orbitofrontal cortex; MT, medial temporal lobe; OC, occipital cortex; O_WM_, occipital white matter; PC, parietal cortex; P_WM_, parietal white matter; PCC, posterior cingulate cortex; pCR, posterior corona radiata; premotor cortex, PMC; sCR, superior corona radiata; SFG, superior frontal gyrus; STC, superior temporal cortex; T_WM_, temporal white matter; thal, thalamus; TC, temporal cortex.

Overall, the exposure to individual HMOs was significantly associated with MRI measures of tissue microstructure and rCBF in the infant brain at 1 month of age. Because the associations of fucosylated and sialylated HMOs with neuroimaging indices for infants in the total sample were the same as the associations of these HMOs with neuroimaging indices for infants of secretor mothers only, we present the results for infants in the total sample. Exposure to 2′FL was associated with reduced FA values throughout the cortex and increased MD values in the posterior cortical gray matter (e.g., right posterior cingulate cortex, PCC; B = 0.02, *p* = 0.001), posterior white matter (e.g., left posterior corona radiata, pCR; B = 0.02, *p* = 0.003), and subcortical gray matter nuclei (e.g., left lenticular nucleus; B = 0.01, *p* = 0.005) (Figure 2). Exposure to 2′FL was associated with reduced rCBF in the cortical gray matter of the frontal, temporal, parietal, and occipital lobes (e.g., superior temporal cortex; B = −0.01, *p* = 0.005) (Figure 2).

### 3.2. HMO 3FL Exposure at 1 Month of Age and Newborn MRI Measures

**Figure 3:**
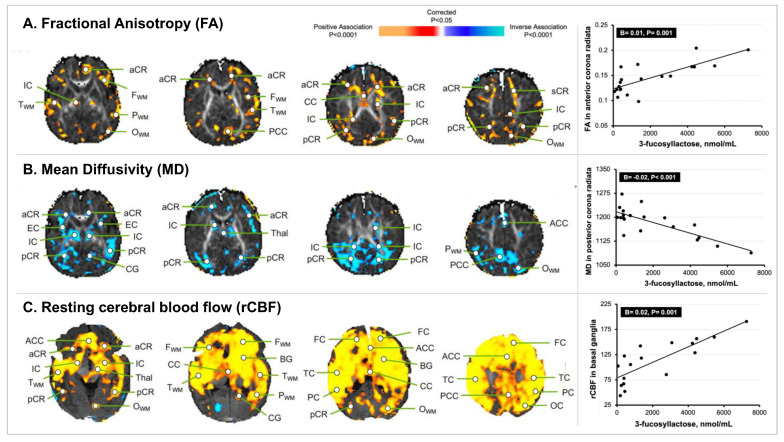
**Statistical maps of 3FL exposure at 1 month of age with newborn MRI measures.** The statistical significance of the associations of 3FL exposure with measures of brain tissue microstructure and rCBF at each point on the surface of the brain is color-coded, with warm colors representing significant positive associations, and cool colors representing significant inverse associations. Only the *p*-values that survived the FDR correction are plotted. (**A**) Concentrations of 3FL positively associated with the FA values in the developing white matter. The scatterplot highlights the significant positive association of 3FL exposure with the FA values in the right aCR. (**B**) Concentrations of 3FL inversely associated with the MD values in similar regions of the developing white matter as those of the FA values. The scatterplot highlights the significant inverse association of 3FL exposure with the MD values in the right pCR. (**C**) Concentrations of 3FL positively associated with the rCBF values throughout much of the cortex and in the developing white matter. The scatterplot highlights the significant positive association of 3FL exposure with the rCBF values in the BG. Abbreviations: ACC, anterior cingulate cortex; aCR, anterior corona radiata; BG, basal ganglia; CB, cerebellum; CG, cingulum; CC, corpus callosum; DLPFC, dorsolateral prefrontal cortex; EC, external capsule; FC, frontal cortex; F_WM_, frontal white matter; IFC, inferior frontal cortex; IOC, inferior occipital cortex; IPC, inferior parietal cortex; IN, insula; IC, internal capsule; LOFC, lateral orbitofrontal cortex; lent, lenticular nucleus; MOFC, medial orbitofrontal cortex; MT, medial temporal lobe; OC, occipital cortex; O_WM_, occipital white matter; PC, parietal cortex; P_WM_, parietal white matter; PCC, posterior cingulate cortex; pCR, posterior corona radiata; premotor cortex, PMC; sCR, superior corona radiata; SFG, superior frontal gyrus; STC, superior temporal cortex; T_WM_, temporal white matter; thal, thalamus; TC, temporal cortex.

Exposure to 3FL was associated with increased FA values in the white matter throughout the frontal, temporal, parietal, and occipital lobes, but particularly in the left internal capsule (IC) and right anterior corona radiata (aCR) (B = 0.01, *p* = 0.001) (Figure 3). Exposure to 3FL was associated with reduced MD values in similar regions (e.g., left IC; B = −0.01, *p* = 0.007) and especially in the posterior white matter (e.g., left pCR; B = −0.02, *p* < 0.001). In addition, exposure to 3FL was associated with increased rCBF in the cortex of most of the brain and within the white matter of the frontal, temporal, parietal, and occipital lobes (e.g., right occipital white matter; B = 0.01, *p* = 0.001) and the subcortical gray matter nuclei (e.g., right basal ganglia; B = 0.02, *p* = 0.001).

### 3.3. HMO 3′SL Exposure at 1 Month of Age and Newborn MRI Measures

**Figure 4:**
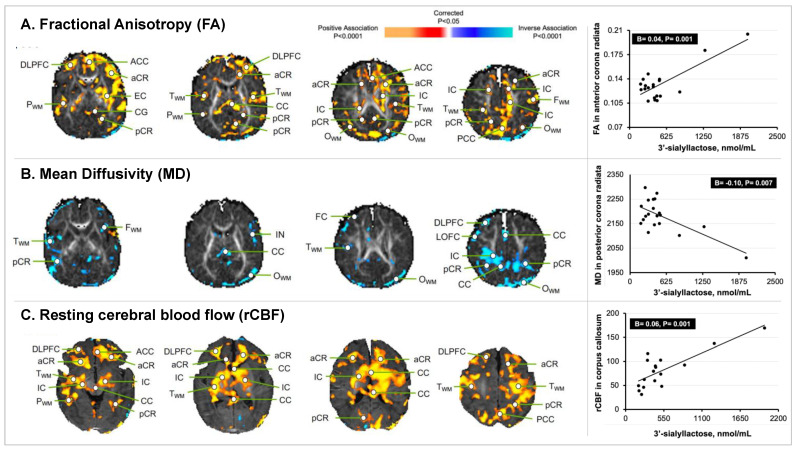
**Statistical maps of 3′SL exposure at 1 month of age with newborn MRI measures.** The statistical significance of the associations of 3′SL exposure with measures of brain tissue microstructure and rCBF at each point on the surface of the brain is color-coded, with warm colors representing significant positive associations, and cool colors representing significant inverse associations. Only the *p*-values that survived the FDR correction are plotted. (**A**) Concentrations of 3′SL positively associated with the FA values in the developing white matter throughout much of the brain and in the cortical gray matter of the frontal lobe. The scatterplot highlights the significant positive association of 3′SL exposure with the FA values in the right aCR. (**B**) Concentrations of 3′SL inversely associated with the MD values in the posterior white matter regions. The scatterplot highlights the significant inverse association of 3′SL exposure with the MD values in the left pCR. (**C**) Concentrations of 3′SL positively associated with the rCBF values in white matter pathways bilaterally and in the cortical gray matter of the frontal lobe; these findings were in similar locations as the associations of 3′SL with the FA values. The scatterplot highlights the significant positive association of 3′SL exposure with rCBF values in the CC. Abbreviations: ACC, anterior cingulate cortex; aCR, anterior corona radiata; BG, basal ganglia; CB, cerebellum; CG, cingulum; CC, corpus callosum; DLPFC, dorsolateral prefrontal cortex; EC, external capsule; FC, frontal cortex; F_WM_, frontal white matter; IFC, inferior frontal cortex; IOC, inferior occipital cortex; IPC, inferior parietal cortex; IN, insula; IC, internal capsule; LOFC, lateral orbitofrontal cortex; lent, lenticular nucleus; MOFC, medial orbitofrontal cortex; MT, medial temporal lobe; OC, occipital cortex; O_WM_, occipital white matter; PC, parietal cortex; P_WM_, parietal white matter; PCC, posterior cingulate cortex; pCR, posterior corona radiata; premotor cortex, PMC; sCR, superior corona radiata; SFG, superior frontal gyrus; STC, superior temporal cortex; T_WM_, temporal white matter; thal, thalamus; TC, temporal cortex.

Exposure to the sialylated HMO 3′SL was associated with increased FA values in the white matter throughout the brain (Figure 4), particularly, in the splenium of the corpus callosum (CC), pCR, and aCR (e.g., right aCR; B = 0.04, *p* = 0.001), and in the cortical gray matter of the frontal lobe, including the anterior cingulate cortex (ACC) and dorsolateral prefrontal cortex (DLPFC) (e.g., right DLPFC; B = 0.03, *p* = 0.001). Exposure to 3′SL was associated with reduced MD values in similar regions, particularly in the posterior white matter (e.g., left pCR; B = −0.10, *p* = 0.007). Exposure to 3′SL was associated with increased rCBF in the white matter bilaterally, including the IC (B = 0.04, *p* < 0.001), CC (B = 0.06, *p* = 0.001), and aCR (B = 0.07, *p* < 0.001), and in the cortical gray matter of the frontal lobe (DLPFC, ACC). In the total sample, the 3′SL value for one participant was identified as a statistical outlier. However, the participant was included in the analysis because the 3′SL value was biologically plausible based on previous reports [22].

### 3.4. Sia Exposure at 1 Month of Age and Newborn MRI Measures

Similar findings were observed for the associations of total HMO-bound Sia exposure with MRI measures. Exposure to total HMO-bound Sia was associated with increased FA values in the white matter throughout the brain and in the cortical gray matter of the DLPFC, ACC, and PCC, and with reduced MD values in the posterior white matter and subcortical gray matter nuclei. Exposure to total HMO-bound Sia was associated with increased rCBF in similar white matter regions, including the IC and the CC, and in the gray matter in the frontal lobe (DLPFC, ACC). In contrast, exposure to the structural isomer 6′SL was not significantly associated with any MRI measures.

## 4. Discussion

To our knowledge, this is the first study to report associations of individual HMOs with MRI indices of infant brain development. We found that candidate HMOs at 1 month of age were each associated with regionally specific DTI- and ASL-derived indices of tissue microstructure and rCBF, suggesting their involvement in specific maturational processes in the cortical gray matter and developing white matter. For example, 2′FL exposure was associated with reduced FA, increased MD, and reduced rCBF values in similar locations within the cortical mantle, suggesting that this HMO is important for the development of dendritic arbors and synapses for circuit formation in the brain. In contrast, 3FL and 3′SL exposures were associated with increased FA, reduced MD, and increased rCBF values in similar regions within the developing white matter, suggesting that these HMOs may enhance the structural connectivity in the brain. We also noted that 6′SL, which is almost structurally identical to 3′SL, had no significant effects.

HMOs are indigestible carbohydrates that include more than 150 distinct structural permutations, and these slight differences may determine diverse physiological roles in brain development [36]. 2′FL is the most abundant HMO in the milk of most mothers and has been shown to influence the characteristics of tissue microstructure and cognition [20]. We previously reported that greater 2′FL exposure at 1 month of age was associated with better cognitive development at 24 months of age [6]. We therefore postulated that 2′FL exposure may influence the development of the brain’s tissue characteristics and neural circuit formation during early infancy, the most rapid period of postnatal brain growth, the time when the brain attains 70% of its adult size [37].

To test this hypothesis and build on the findings of our prior study, we used MRI to examine the influence of 2′FL exposure on brain development in the same cohort of infants, but at 1 month rather than 24 months of age. We found that 2′FL exposure was inversely associated with the FA values throughout most of the cortical mantle. It was also positively associated with the MD values in the posterior portions of the developing white matter. Prior human studies have shown that the FA values in the cortical mantle decline with age during late gestation [38]. Animal studies have shown that this decline in FA values is accompanied by increasing MD values and derives from an age-related increase in dendritic arborization within the cortical mantle [39]. FA declines, and MD increases with increasing dendritic arborization in the cortical gray matter because dendrites and axons arborize without directional preference [40]. Our findings therefore suggest that 2′FL exposure at 1 month of age increases the dendritic arborization throughout the cortical mantle. Dendritic arborization and synaptogenesis are the microstructural basis for learning and memory. Increased dendritic arborization and synapse formation could therefore account for the improved cognitive outcomes associated with 2′FL exposure found in this same cohort at 24 months of age [6].

2′FL exposure was also inversely associated with rCBF in the gray matter of the frontal, temporal, and parietal cortices, in similar locations where we observed inverse associations of 2′FL with the FA values. Because rCBF is tightly coupled with cellular metabolism [41,42,43], it is considered a valid surrogate measure of the local metabolism. Reduced rCBF in the cortical gray matter therefore represents a lower metabolism. The presence of a lower metabolism in the same cortical locations of greater dendritic arborization and synaptic density suggests that the neural circuits forming in early infancy may be metabolically more efficient in direct proportion to the quantity of 2′FL in the milk consumed during early infancy. The formation of more efficient neural circuits would account for the improved cognitive outcomes associated with 2′FL ingestion in this same cohort of infants. The possibility that 2′FL promotes the formation of more efficient neural circuits and thereby improves cognitive development is consistent with the widely held theory that metabolically more efficient neural circuits are the cellular basis for higher intelligence [44,45].

We also found that 3FL and 3′SL exposures were positively associated with the FA values in the developing white matter. They were also inversely associated with the MD values in these same general locations. Studies in infants have shown that the FA values in developing axonal tracts increase rapidly with age from birth to 12 months [40] due to an age-related increase in myelination that improves the white matter integrity [46]. As the FA values increase in the developing axonal tracts, the MD values decrease in these same locations because myelination, as well as changes in axon density and orientation, constrain the movement of water parallel to the long axis of nerve fiber bundles. Myelination supports faster information processing and improved information transfer throughout the brain, critical for higher-order cognitive functions [47].

We found that w3FL and 3′Sere L also positively associated with rCBF values in the developing white matter in similar locations where we observed their positive associations with the FA values, consistent with findings in adults [48]. The FA and MD associations that suggest 3FL and 3′SL may promote a more rapid myelination in the developing white matter therefore also suggest that the concomitant associated increase in rCBF in these white matter regions may be driven by a significant metabolic demand in oligodendrocytes, which produce myelin [49]. The interpretations of these findings underscore the value of acquiring multiple imaging modalities in the same brains. Each modality provides unique information about tissue characteristics at each brain location, and their use in combination better constrains the interpretation of possible cellular determinants for the observed effects [50,51,52,53]. Of note, we did not observe significant associations of the HMO 6′SL exposure with MRI measures of brain development in infants, suggesting that not all HMOs contribute significantly to a given neurodevelopmental outcome.

The identification of 3FL and 3′SL as having distinct roles in brain development at 1 month of age is particularly relevant, given our recent study finding that 3FL and 3′SL were the only HMOs to increase across lactation. In our comprehensive analysis of HMO changes over lactation, most HMO concentrations declined sharply within the first 6 months. We also found that 2′FL did not significantly change over 24 months, while only two other HMOs significantly increased over time, i.e., 3FL (10-fold increase) and 3′SL (2-fold increase) [54].

Collectively, our results suggest that specific HMOs may influence the tissue microstructure in the infant brain through several mechanisms. HMOs are prebiotics for the gut microbiome which produces metabolites that may affect the early brain development. Treatment with 2′FL and 3FL in vitro increased the abundance of *Lactobacillus* and *Bacteroides* [10], gut microbes that have been associated with better cognitive development in infants [55]. A greater abundance of *Lactobacillus* and *Bacteroides* produced greater concentrations of short-chain fatty acids (SCFAs [10], metabolites that cross the blood–brain barrier and are used as energy substrates to support the cellular metabolism during early brain development) [56]. SCFAs also affect cell signaling and the induction of tyrosine hydroxylase that increases neurotransmitter synthesis and release [57,58]. These prior studies suggest that 2′FL and 3FL enhance the growth of distinct gut bacteria, which produce SCFAs and other metabolites that may support more rapid dendritic arborization, synaptogenesis, and myelination.

Another potential mechanism whereby HMOs may produce the observed associations with MRI measures is by lending a structural support for maturational events in the newborn brain. The sialylated HMOs 3′SL and 6′SL contain SA, an essential component of brain gangliosides and of the polysialylated neural cell adhesion molecule (NCAM) [19,59]. Gangliosides and polysialylated NCAM play critical roles in cell-to-cell interactions, neuronal outgrowth, neuronal migration, and the modulation of synaptic activity, ultimately supporting improved memory formation [59]. For example, piglets fed 3′SL, 6′SL, and SA alone performed better on learning and memory tasks compared to controls, which was attributed to increased ganglioside concentrations in the prefrontal cortex and corpus callosum [8,9]. We observed the associations of 3′SL and total HMO-bound Sia exposures with newborn MRI measures in similar locations of the prefrontal cortex and corpus callosum. Gangliosides and polysialylated NCAM are also receptors for myelin-associated glycoprotein expressed on the innermost myelin membrane adjacent to the axon’s surface, where it enhances axon–myelin stability [60]. Exposure to 3′SL and total HMO-bound Sia may therefore enhance gangliosides and polysialylated NCAM production for axon–myelin interactions and axon stability, accounting for the observed 3′SL and total HMO-bound Sia associations with MRI indices of white matter maturation in our sample. However, the structural isomer 6′SL, which also carries SA in a different linkage, was not associated with MRI measures, indicating that the effects are not simply based on the provision of SA, but are in fact structurally dependent.

This study has several limitations. The use of a cross-sectional design with a one-time measurement of individual HMO exposures and brain MRI measures limits causal interpretations. Other limitations include a poor generalizability, as our findings are specific to a small sample of Hispanic mother–infant dyads from lower socioeconomic households in the Southwestern United States. The limited number of participants also reduced our statistical power to detect true associations and could contribute to the detection of spurious findings. We did not collect information on the infant’s intake of dietary supplements (e.g., probiotics, fat-soluble vitamins), which could conceivably affect the gut microbiome of the infants [61,62,63], thereby influencing the proposed mechanism through which HMOs may be associated with MRI indices of early brain development. We also did not report the associations of individual HMO exposures with neurodevelopmental outcomes in this subset of infants, although we note that this was the focus of our prior publication and will also be examined in future studies in older infants [6].

## 5. Conclusions

In conclusion, our data show that individual HMO exposures were differentially associated with MRI indices of newborn brain tissue organization and rCBF, which reflect the structural and metabolic characteristics that foster future cognitive and behavioral functions. Our findings highlighted specific roles of 2′FL, 3FL, and 3′SL in promoting the maturation of the cortical gray matter and the developing white matter, which in turn may guide recommendations for the nutritional care of infants and supplementation strategies that support optimal brain development. Although 2′FL has been introduced as an ingredient in commercial formula for term infants, our findings suggest that the addition of 3FL and 3′SL could also be beneficial, given their distinct yet complementary influences on early brain tissue organization.

## Figures and Tables

**Table 1:** Characteristics of the participants and their infants ^1^.

Variable	Mean	SD	Min.	Max.
Mothers				
Age at delivery (years)	28.5	6.74	18	40
Pre-pregnancy BMI (kg/m^2^)	27.8	6.25	18.7	42.5
Secretor status (%)	85.0			
Education level (%)				
Less than high school	20.8			
Completed high school	58.4			
Completed college	20.8			
Infants				
Female (%)	62.5			
Birthweight (g)	3360	498	2300	4510
Gestational age at birth (weeks)	39.5	1.03	37.0	41.1
Postmenstrual age at MRI scanning (days) ^2^	323	10.5	300	351
Breast feedings per day, 1-month (number)	7.08	1.64	1	8

^1^ Values are mean ± SD or %. ^2^ Postmenstrual age at the time of MRI scanning is defined as the time that elapsed between the mother’s last menstrual period and the birth of the infant (i.e., gestational age at birth) plus the chronological age of the infant from birth to the time of the MRI scan [35].

## Data Availability

Data described in the manuscript, code book, and analytic code will be made available upon request pending application and approval from the authors.

## Supplementary Materials

The following supporting information can be downloaded at: https://www.mdpi.com/article/10.3390/nu14183820/s1, File S1: Materials and Methods.
