# Supplementary material for: Associations of Human Milk Oligosaccharides with Infant Brain Tissue Organization and Regional Blood Flow at 1 Month of Age
Source: Nutrients. 2022 Sep 16;14(18):3820. doi: 10.3390/nu14183820 (PMC9501015; doi:10.3390/nu14183820)
Supplement: Supplementary file 1 [file nutrients-14-03820-s001.zip › nutrients-1843331-supplementary.pdf]

SUPPLEMENTARY MATERIAL TABLE OF CONTENTS

Materials and Methods

MRI scanning procedures…………………………………………………………………2

MRI pulse sequences…………………………………………………………………………3

MRI data processing………………………………………………………………………..4

ASL data processing…………………………………………………………………………5

References……………………………………………………………………………………...6

SUPPLEMENTARY MATERIAL: MATERIALS AND METHODS

MRI scanning procedures

For each infant, we used a rigid body transformation to coregister all diffusion weighted images (DWIs) to their B0 image. We used the 3 translation and 3 rotation parameters from that transformation to calculate 2 head motion indices: (1) the Root Mean Squared (RMS), which computes the root of the mean squared displacement from a head represented as a 50 mm radium sphere; and (2) Mean Framewise Displacement (FD), which convers rotational angular displacements to translational displacements for the surface of a 50 mm radius sphere. Images with >0.5 mm motion estimated by either RMS or FD were removed from further preprocessing; if >10% of images had this much motion, that entire DTI dataset would be excluded from further processing. Next, we used quadratic warping along the anterior-posterior direction to correct spatial distortions induced by eddy currents in the phase-encoding direction. We also visually assessed motion by constructing tensor color maps from the retained images and displaying the principal eigenvectors throughout the brain, which show a color bias in the presence of motion artifact. Infants with motion artifacts were excluded from analyses (n=4).

MRI pulse sequences

Multi-Shell DTI. DTI data were acquired in oblique slices parallel to the AC-PC line using a multiband, single-shot spin echo (SE) imaging sequence with matrix=128x128, TR=5300ms, TE= 89 ms, FOV=24cm, Flip=78°, 72 axial oblique slices, Slice thickness=2.0 mm, Slice Spacing=0 mm; EPI factor=119, PFOV=1.0, multiband factor=3. Three baseline images were acquired with b=0 s/mm^2 and 100 diffusion weighted images (DWIs) with multishell b=500, 1000, 2000, and 3000 s/mm^2 along directions that were sampled uniformly.

2D Pseudo Continuous ASL (2D pCASL) We used a single-shot, echo planar imaging (EPI) sequence for image acquisition, with TR=4550ms, TE=13ms, Flip Angle = 90°, EPI factor=39, post labeling delay (PLD)=1525ms, 2 background suppression pulses, labeling delay (LD)=1800 ms, FOV=24x24 cm^2, 28 axial oblique slices, inter slice spacing=0mm, slice thickness=5mm, in-plane resolution = 2.75x2.75mm^2, number of volumes acquired = 60. For quantification of regional cerebral blood flow (rCBF), we acquired an M_0 image along the same slices as 2D pCASL data using a multislice, turbo spin echo (TSE) sequence with TR=7000 ms, TE=9 ms, Flip Angle = 90°, SENSE factor=2, FOV=24x24cm^2, 28 axial oblique slices, inter slice spacing = 0 mm, slice thickness=5mm, in-plane resolution=2.75x2.75mm^2.

Anatomical High-Resolution T1-Weighted Images T1-weighted (T1w) anatomical images were acquired to aid spatial coregistration of the DTI and ASL datasets. They were acquired using a 3D Gradient Echo (GE) sequence with inversion recovery: repetition time (TR)=6.3 ms, echo time (TE)=2.9 ms, Turbo Fast Echo (TFE) pre-pulse = 1060 ms, flip angle=8°, matrix=256x256, field of view=25 cm, phase field of view=100%, slice thickness=1.0 mm, TFE factor=170, number of sagittal slices=170, voxel size=1x1x1mm3. Two images each of NEX=1 was acquired and then averaged offline.

MRI data processing

Selection of the Template Brain We used a single representative brain for the T1-weighed template, rather than one derived by averaging brains across multiple infants, because a single brain has well-defined tissue interfaces, including those at CSF-gray matter or gray-white matter interfaces to improve the precision of spatial co-registration.[1-3] We first identified, as a preliminary template, the brain of one infant whose postmenstrual age (PMA) at the time of scan and overall brain size were nearest the group averages. The brains for all remaining infants in the sample were coregistered to that preliminary template using a rigid body transformation followed by a high-dimensional, nonrigid warping algorithm based on the principles of fluid-flow dynamics [4], which permitted point-to-point matching of the brain surface for each infant with the surface of the template brain. The brain for which all points across its surface were closest (in the least squares sense) to the average of the distances across those points for the entire sample was selected as the final template, thereby yielding a template brain that was specific to and morphologically most representative of all brains in this cohort.

Maps of DTI Scalar Indices We estimated the diffusion tensor (D) at each voxel of the pre-processed DTI data in DSI Studio (RRID:SCR_009557). We ensured that the fitted tensor D was positive definite by first decomposing the tensor as the product D = A*AT and estimating the matrix A, then using the estimated matrix $\tilde{A}$ to compute the positive definite tensor $\tilde{D}=\tilde{A}*\tilde{A}T$. We decomposed this positive definite tensor into its eigenvalues (λ1, λ2, λ3) and eigenvectors (v1,v2,v3), which we then used to compute the scalar indices fractional anisotropy (FA), mean diffusivity (MD), axial diffusivity (AD), and radial diffusivity (RD):

$$FA = \frac{\sqrt{(\lambda_1-\lambda_2)^2+(\lambda_2-\lambda_3)^2+(\lambda_3-\lambda_1)^2}}{\sqrt{2*(\lambda_1^2+\lambda_2^2+\lambda_3^2)}}, MD = \frac{1}{3}(\lambda_1+\lambda_2+\lambda_3), RD = \frac{1}{2}(\lambda_2+\lambda_3), \text{ and } AD = \lambda_1$$

FA quantifies the degree of directional diffusion of water, which is used as an index of local tissue organization and integrity of white matter fiber tracts. MD represents the directionless magnitude of water diffusion. AD and RD measure the magnitude of water diffusion in the direction parallel and perpendicular, respectively, to the primary axis of the diffusion tensor. When considered together, FA, MD, RD, and AD maps aid interpretation of the biological basis for DTI findings.

ASL data processing

Using a single compartment model, the perfusion (f) was calculated as:[5] $f = \frac{6000 \cdot \Delta M \cdot e^{PLD/T_{1a}} \cdot e^{TE/T_{2a}^{*}}}{2\alpha \cdot \alpha_{inv} \cdot M_{0a} \cdot T_{1a}\left(1 - e^{-\tau/T_{1a}}\right)}$, where ΔM is the difference of the control and the label images, M_{0a} is the equilibrium magnetization of arterial blood, PLD=1,525 ms is the post labeling delay, $T_{1a} = 1.8$ sec is the longitudinal relaxation time of the arterial blood, $T_{2a}^{*} = 50$ ms is the transverse relaxation time of the arterial blood, $\alpha = 0.8$ is the labeling efficiency, $\alpha_{inv} = 0.83$ is the correction for the labeling efficiency due to 2 background suppression pulses.[6] The equilibrium magnetization of the arterial blood M_{oa} was estimated by multiplying the equilibrium magnetization of the CSF M_{oCSF} with the blood-water partition coefficient of 0.76 ml water/ml water[7,8] and density of brain tissue (1.05 g/ml).[9]

REFERENCES

1. Plessen KJ, Bansal R, Zhu H, et al. Hippocampus and amygdala morphology in attention-deficit/hyperactivity disorder. *Arch Gen Psychiatry.* 2006;63(7):795-807.

2. Amat JA, Bansal R, Whiteman R, Haggerty R, Royal J, Peterson BS. Correlates of intellectual ability with morphology of the hippocampus and amygdala in healthy adults. *Brain Cogn.* 2008;66(2):105-114.

3. Bansal R, Staib L, Xu D, Zhu H, Peterson B. Statistical analyses of brain surfaces using Gaussian random fields on 2-D manifolds. *IEEE Trans Med Imaging.* 2007;26:46-57.

4. Christensen G, Joshi, S., Miller, M. Volumetric transformation of brain anatomy. *IEEE Trans Med Imaging.* 1997;16(6):1369-1383.

5. Alsop DC, Detre JA, Golay X, et al. Recommended Implementation of Arterial Spin-Labeled Perfusion MRI for Clinical Applications: A Consensus of the ISMRM Perfusion Study Group and the European Consortium for ASL in Dementia. *Magnetic Resonance in Medicine.* 2015;73(1):102-116.

6. Mutsaerts HJMM, Steketee RME, Heijtel DFR, et al. Inter-Vendor Reproducibility of Pseudo-Continuous Arterial Spin Labeling at 3 Tesla. *PLoS One.* 2014;9(8).

7. Herscovitch P, Raichle ME. What Is the Correct Value for the Brain Blood Partition-Coefficient for Water. *J Cerebr Blood F Met.* 1985;5(1):65-69.

8. Heijtel DFR, Mutsaerts HJMM, Bakker E, et al. Accuracy and precision of pseudo-continuous arterial spin labeling perfusion during baseline and hypercapnia: A head-to-head comparison with O-15 H2O positron emission tomography. *NeuroImage.* 2014;92:182-192.

9. Chalela JA, Alsop DC, Gonzalez-Atavales JB, Maldjian JA, Kasner SE, Detre JA. Magnetic resonance perfusion imaging in acute ischemic stroke using continuous arterial spin labeling. *Stroke.* 2000;31(3):680-687.
